# Pathological and Incidental Findings on Brain MRI in a Single-Center Study of 229 Consecutive Girls with Early or Precocious Puberty

**DOI:** 10.1371/journal.pone.0029829

**Published:** 2012-01-12

**Authors:** Signe Sloth Mogensen, Lise Aksglaede, Annette Mouritsen, Kaspar Sørensen, Katharina M. Main, Peter Gideon, Anders Juul

**Affiliations:** 1 Department of Growth and Reproduction, Rigshospitalet, Faculty of Health Sciences, University of Copenhagen, Copenhagen, Denmark; 2 Department of Radiology, Rigshospitalet, Faculty of Health Sciences, University of Copenhagen, Copenhagen, Denmark; Institution of Automation, CAS, China

## Abstract

Central precocious puberty may result from organic brain lesions, but is most frequently of idiopathic origin. Clinical or biochemical factors which could predict a pathological brain MRI in girls with CPP have been searched for. With the recent decline in age at pubertal onset among US and European girls, it has been suggested that only girls with CPP below 6 years of age should have brain MRI performed.

**Objective:**

To evaluate the outcome of brain MRI in girls referred with early signs of puberty in relation to age at presentation as well as clinical and biochemical parameters.

**Method:**

A single-center study of 229 consecutive girls with early or precocious puberty who had brain imaging performed. We evaluated medical history, clinical and biochemical factors, and four groups were defined based on the outcome of their MRI.

**Results:**

Thirteen out of 208 (6.3%) girls with precocious puberty, but no other sign of CNS symptoms, had a pathological brain MRI. Importantly, all 13 girls were above 6 years of age, and 6 girls were even 8–9 years old. Twenty girls (9.6%) had incidental findings on brain MRI. Furthermore, 21 girls had known CNS pathology at time of evaluation. Basal LH was significantly higher in girls with newly diagnosed CNS pathology compared to girls with a non-pathological MRI (p = 0.025); no cut of value was found as values overlapped.

**Conclusion:**

A high frequency of 6–8 year old girls with precocious puberty in our study had a pathological brain MRI, which could not be predicted from any clinical nor biochemical parameters. Thus, we believe that girls with precocious pubertal development of central origin before 8 years of age should continue to be examined by a brain MRI.

## Introduction

True precocious puberty in girls is defined by the development of secondary sexual characteristics before the age of 8 years [1]–[3], and is accompanied by growth acceleration, advancement of bone age and elevated sex steroid levels. Precocious puberty secondary to the activation of the hypothalamic-pituitary-gonadal axis (gonadotropin-dependent) is termed central precocious puberty (CPP). Recent studies find that only a minority of cases with CPP in girls is caused by central nervous system (CNS) abnormalities, i.e. organic CPP (OCPP) [4]–[11]. Importantly, *magnetic resonance imaging* (MRI) has until today been performed to rule out brain abnormalities in all girls with CPP. The most frequently detected brain abnormalities associated with CPP include hypothalamic hamartomas, optic gliomas, astrocytomas, pineal tumours, post-infectious encephalitis, hydrocephalus, neurofibromatosis and previous CNS injury [6], [9], [12], [13].

Recent studies have evaluated clinical and biochemical predictors to ensure early identification of girls with brain abnormalities. A French study of 197 girls with precocious puberty from a single-center suggested that chronological age lower than 6 years or the combination of chronological age older than 6 years and a serum concentration of estradiol (E2) above 45 pmol/L was predictive of OCPP [14]. This algorithm was verified in a European multi-center cohort study which included 443 girls from seven different centers, although hormone assays varied among centers [8].

In a recent study of Caucasian girls referred with early signs of puberty, we found that levels of basal luteinizing hormone (LH) could partly predict the outcome of GnRH-testing. Furthermore, we found that estradiol levels were above +2 SD in 86% of girls with OCPP compared to 46% of girls with idiopathic CPP [4]. So far, no single clinical or biochemical predictor of a pathological brain MRI in girls with CPP has been identified, although young age, rapid progression and high estradiol levels are considered factors which may predict higher risk of brain abnormalities [3], [14]. Also body mass index (BMI), bone age advancement, peak LH, peak follicle-stimulating hormone (FSH) and LH/FSH levels have been proposed as factors which may identify girls at high risk of OCPP [7]. Though, the concern remain by the potential risk of missing treatable brain abnormalities from leaving out MRI in low risk girls, including 6–8 year old girls [5], [7], [10], [15], [16]. The recent downwards changes in age at pubertal onset in US and Europe [17], [18] may theoretically challenge the appropriate age limit from which girls with CPP should have a brain MRI performed.

The aim of this study is to evaluate the outcome of brain MRI in 229 consecutive girls referred with early signs of puberty, and to evaluate possible predictive factors in girls with pathological brain MRI outcome.

## Methods

### Patient population

We included girls referred because of clinical signs of early or precocious puberty to our tertiary referral university department at Rigshospitalet in Copenhagen, Denmark, during a 16-year period from January 1^st^ 1993 to January 31^st^ 2009. Patients were recorded in our centralized register upon referral with a diagnosis according to the World Health Organization's International Classification of Diseases, Tenth Revision (ICD-10) in our department. The referral diagnoses included central precocious puberty (DE288A), premature thelarche (DE308A), premature adrenarche (DE270B) and early-normal pubertal variant (DE301). To make sure we included all girls referred with precocious puberty we also included girls primarily coded with an observational diagnosis code (DZ038 or DZ003) in combination with an additional ICD-10 code related to early puberty. We evaluated the girls with pubertal onset before 9 years of age based on available reference data from 1991 on pubertal timing in healthy Danish girls [19]. Furthermore, to complete the cohort and make this study clinically representative, we included girls referred to our department with pubertal onset even after the age of 9 as previously described [4]. Altogether 786 consecutive girls were referred of whom 47 patient's files were lost to follow up. Of the 739 girls referred for evaluation, 239 had imaging of the brain performed. Out of these 239 girls, 10 girls were excluded because of insufficient data resulting in a cohort of 229 girls.

### Hormone analyses

Non-fasting venous blood samples were obtained from all girls to determine FSH, LH and estradiol. All reproductive hormones were determined in duplicates in our own hormone laboratory using the same methods during the entire study period. LH and FSH were measured by ultrasensitive time-resolved immunofluorometric assays (Delfia®, Wallac, Inc., Turku, Finland) [20]. The detection limits for LH, FSH were 0.05 IU/L and 0.06 IU/L, respectively. Estradiol was measured by radioimmunoassay with a detection limit of 18 pmol/l (Pantex, Santa Monica, CA [before 1998 distributed by Immuno Diagnostic System, Bolden, United Kingdom]) [20].

### Clinical examination

The clinical examination included a full history from the patient and her family, followed by an examination of pubertal development according to the Marshall and Tanner criteria [21], including both inspection and palpation of glandular breast tissue. Height and weight were measured, BMI was calculated as weight/height^2^, and the respective standard deviation scores (SDS) were calculated based on Danish reference data [22], [23]. Bone age was assessed by X ray of left hand according to Greulich and Pyle [24].

### GnRH test

A gonadotropin-releasing hormone (GnRH) stimulation test was performed in 188 girls. Serum LH and FSH were determined 0 and 30 minutes after the intravenous bolus administration of GnRH (100 µg Relefact, Sanofi-Aventis, Frankfurt am Main, Germany). In accordance with guidelines [3], we defined CPP by: 1) peak LH≥5 IU/L or 2) stimulated FSH/LH ratio ≥0.66 IU/L or 3) if GnRH test was not performed the hormone level was considered pubertal if basal LH levels above 0,3 IU/L according to Neely et al. [25].

### Brain magnetic resonance imaging

All girls included had brain imaging performed by magnetic resonance imaging (MRI) (n = 207) or alternatively by computed tomography (CT) (n = 22) performed. CT was made when contraindications were present (e.g. presence of cochlea transplants or claustrophobia). The MRI findings were categorized into 4 groups; 1) well known brain aetiology at time of referral, 2) newly diagnosed CNS pathology, including girls with early or precocious puberty and no other CNS symptom, 3) incidental findings, not associated to precocious puberty, 4) if the MRI showed normal anatomy of the hypothalamic region and no incidental findings in general, it was grouped as normal MRI.

#### Ethical considerations

The part of study regarding the healthy children serving as controls was approved by the ethical committees of the Capital Region (KF 01 282214 and V200.1996/90) and conducted in accordance with the second Helsinki Declaration. All children and guardians gave their informed written consent to the study. The clinical part of the present study is a retrospective evaluation of clinical information from case record files that does not involve renewed contact with the patients, and must in accordance with Act 2003-05-28 no. 402 be defined as a retrospective study, solely entailing registration of specific data regarding the patient. Parents of the patients (all <18 yrs) gave their informed written consent to the diagnostic procedures and subsequent treatment.

### Statistical analyses

The statistical analyses were made using the SPSS software (Version 17.0; SPSS, Inc., Chicago, IL). Diagnostic groups were compared using nonparametric Mann-Whitney U test, and a probability value of p≤0.05 was considered statistically significant.

## Results

Brain MRI was performed in 229 girls. Cerebral findings were abnormal in 54 patients (Table 1). Twenty one girls had well known brain pathologies at the time of referral. In the remaining 208 girls with early or precocious puberty, who had no CNS symptoms, MRI revealed CNS abnormalities in 33 girls. In 13 girls, the newly found MRI abnormalities (6.3%) were considered to be causally related to the early puberty, and in another 20 girls (9.6%) MRI findings were considered as incidental findings, which were not related to the early puberty. The newly diagnosed pathological findings included: arachnoid cysts (n = 5), of which one had hydrocephalus as well, hypothalamic hamartoma (n = 1), pilocytic astrocytoma (n = 1), hydrocephalus and corpus callosum agenesia (n = 1), hamartomas (basal ganglia, brainstem, cerebellum) and neurofibromatosis type 1 (NF1) (n = 1), pineal tumour (n = 1), Chiari II malformation (n = 1), cortical dysplasia in left occipital lobe (n = 1) and pontine tumour (n = 1).

**Table 1:** Anatomical description of 54 brain MRI with abnormal findings.

Newly diagnosed CNS pathologies	13	Known CNS ethiologi	21	Incidental findings	20
Arachnoid cysts	5	Chiari II malformations and MMC	4	Pineal cysts	5
Hamartomas	2	Hamartoma/glioma and NF1	3	Pituitary microadenomas	4
Pilocytic astrocytoma	1	Astrocytomas	3	Pituitary enlargement	3
Hydrocephalus and corpus callosum agenesia	1	Encephalitis sequelae	2	Pituitary enlargement	2
Pineal tumour	1	Hydrocephalus	2	Asymmetric pituitary	1
Chiari II malformation	1	Meningitis sequelae	1	Absent septum pellucidum	1
Cortical dysplasia in left occipital lobe	1	Arachnoid cyst and septo-optic dysplasia	1	Variation of perivascular space (normal)	1
Pontine tumour	1	Traumatic CNS injury	1	Unspecific white matter lesion	1
		Brainstem tumour	1	Hyperintense thalamic lesion	1
		Germinoma	1	Bone proces (clivus)	1
		Porencephalic congenital cyst, hydrocephalus	1		
		Insult in left temporoparital region	1		

### Girls with newly diagnosed CNS pathology

**Figure 1:**
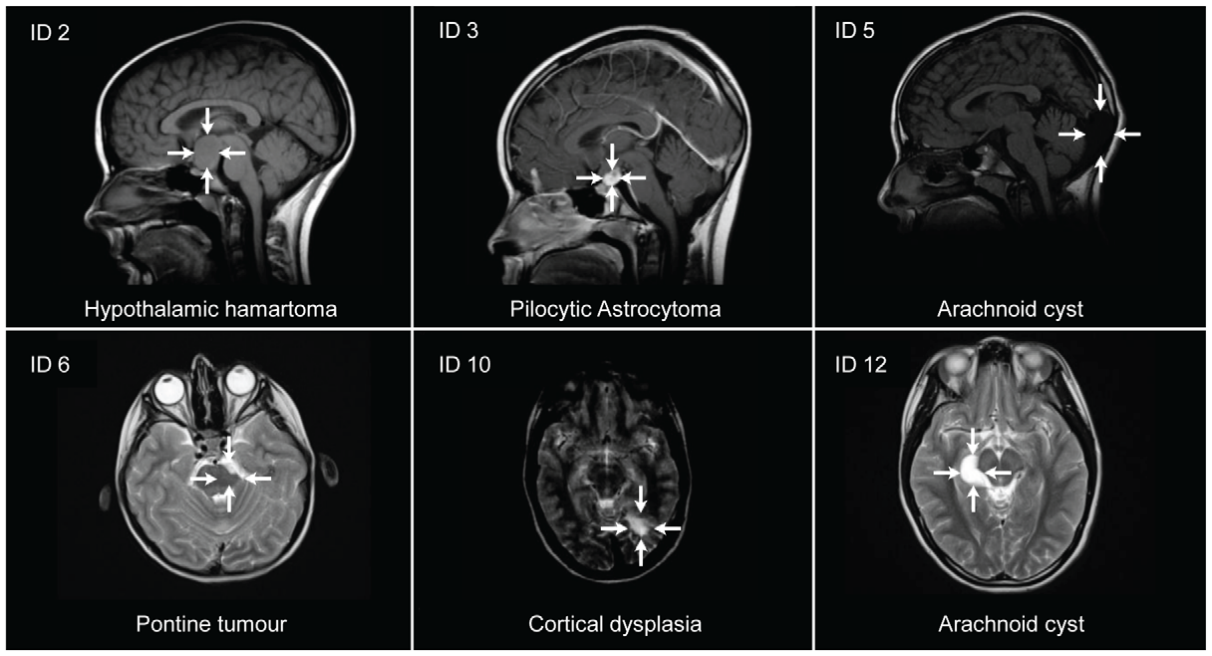
Brain MRI of newly diagnosed pathology. Pathological brain MRI scans from 6 representative girls with precocious puberty and no other CNS symptoms. ID indicating their identification number (see Table 2).

As shown in Table 2, nine of the 13 girls with newly diagnosed brain pathology were treated with GnRH agonists, whereas treatment was not indicated in three of the girls, and one did not receive therapy due to parental decline. Ten girls had two or more MRI scans performed during follow up. The three girls who were scanned only once had congenital malformations (Chiari II malformation, corpus callosum agenesia) or hamartomas in relation to a newly diagnosed NF1, and had clinical follow up in our department or in other paediatric departments. One girl with a pilocytic astrocytoma was operated due to tumour growth, which necessitated performing a hypophysectomy resulting in panhypopituitarism. Another girl, with hypothalamic hamartomas, is still being observed by repeated MRI scans not revealing tumour growth. Representative MRI scans are illustrated in Figure 1.

**Table 2:** Descriptive characteristics of 13 girls with newly diagnosed brain pathology.

ID	CPP according to criteria	MRI findings	Age at onset (years)	Age at 1^st^ exam (years)	Pubertal stage	Height (SDS)	Weight (SDS)	BMI (SDS)	BA-CA (years)	Basal FSH (IU/L)	Peak FSH (IU/L)	Basal LH (IU/L)	Peak LH (IU/L)	Peak LH/FSH ratio	E2 (pmol/L)	GnRH agonist therapy	Clinical Outcome
1	y	Chiari II malformation	6.2	7.9	B3	0,7	ND	ND	3,1	4,45	11,9	1,54	25,4	2.13	53	y	clinical follow up
2	y	Hypothalamic hamartoma	6.4	11.0	B4	0,9	0,8	0,9	2.5	1,6	ND	2,34	ND	ND	611	n	MRI follow up
3	y	Pilocytic astrocytoma	6.5	6.9	B3	1.3	0.8	0.2	−1	2,31	8,94	0,15	13,3	1.48	36	y	Operation
4	n	Hamartomas (basal ganglia, brain stem, cerebellum), NF type 1	7.0	7.7	B1	1,1	1,3	1,1	1,3	1,73	5,62	0,06	1,56	0.28	18	n	clinical follow up
5	y	Arachnoid cyst	7.5	9.9	B3	1,5	2,4	1,5	3,1	2,12	ND	1,13	ND	ND	227	n	MRI follow up
6	y	Pontine tumor	7.5	7.9	B2	1,4	1,9	1,5	2,5	1,8	5,32	0,72	10,7	2.01	30	y	MRI follow up
7	y	Hydrocephalus and arachnoid cyst	7.6	8.0	B2	0,7	1,6	1,3	−0,1	3.77	9.47	0.89	12.5	1.32	61	y	MRI follow up
8	n	Arachnoid cyst	8.2	9.0	B4	1,4	4,1	2,8	2,0	4,39	ND	3,97	ND	ND	47	y	MRI follow up
9	n	Hydrocephalus and corpus callosum agenesia	8.3	9.7	B3	−2	0,1	1,6	1,3	5,79	17	5,47	63,3	3.72	100	y	clinical follow up
10	n	Congenital dysplasia in left occipital lobe	8.5	9.5	B5	0,3	0,8	1	1,5	5,43	6,77	4,4	21,3	3.15	184	y	MRI follow up
11	n	Arachnoid cyst	8.5	9.0	B2	2,2	1,2	0,1	1,7	8,56	14,8	1,41	28,5	1.93	62	y	MRI follow up
12	n	Arachnoid cyst	8.5	9.1	B2	0,9	2,1	1,8	0,5	2,28	8,69	0,12	8,85	1.02	18	n*	MRI follow up
13	n	Pineal tumor	8.5	9.5	B3	1,4	ND	ND	0.5	5,03	ND	1,18	ND	ND	160	y	MRI follow up

y: yes, n:no, ND: Not Determined,

*: indicated but parental decline.

### Girls with incidental CNS findings

**Figure 2:**
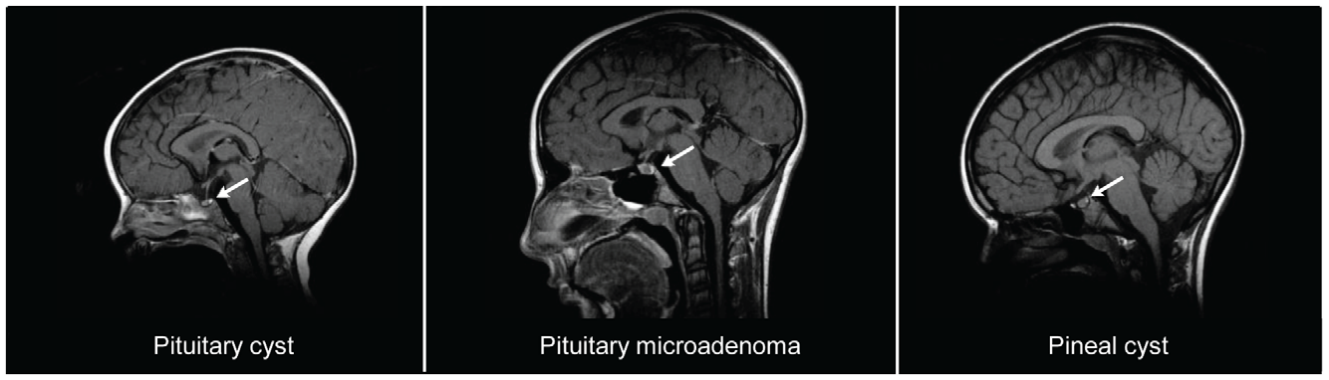
Brain MRI scans of girls with incidental findings. Typical incidental MRI findings in the CNS from three representative girls with early or precocious puberty.

Twelve of the girls were treated with GnRH analogs. Twelve out of the 20 girls with incidental findings had two or more MRI scans during follow up. None of the girls underwent surgery. Representative MRI scans are illustrated in Figure 2.

### Girls with known CNS aetiology

The girls referred with known aetiology showed a broad spectrum of pathologies related to precocious puberty which include chiari malformation and myelomeningocele (MMC) (n = 4), hamartomas/gliomas and NF1 (n = 3), astrocytomas (n = 3), encephalitis sequelae (n = 2), hydrocephalus (n = 2) and another 7 girls with various abnormalities (see Table 1).

### Clinical and biochemical predictors of a pathological MRI

As shown in Table 3 medical history, clinical and biochemical factors were compared within the groups. In the total cohort, 161 girls had pubertal onset before 8 years whereas 68 girls were older than 8 years at pubertal onset, resulting in a median age for pubertal onset of 7.7 years (range 0.4 to 10.0). Median ages at pubertal onset were 7.6 years (range 6.2 to 8.5), 7.7 years (range 2.5 to 10.0), 7.5 years (range 1.0 to 9.5) and 7.7 years (range 0.4 to 9.1) in girls with newly diagnosed CNS pathology, known CNS abnormalities, incidental CNS findings and with a normal MRI, respectively. Six girls with newly diagnosed CNS pathology had pubertal onset after 8 years of age.

**Table 3:** Comparison of clinical and biochemical characteristics girls according to their brain MRI findings.

	I Non-pathological		II CNS Pathology		IIb vs Ia	II vs I	IIb vs I
	Ia Normal MRI	Ib Incidental CNS abnormalities	IIa Known CNS abnormalities	IIb Newly diagnosed CNS Pathologies	p-value	p-value	p-value
**n**	175	20	21	13			
**Age at onset**	7,7 (0,4 to 9,1)	7.5 (1.0 to 9.5)	7,7 (2,5 to 10,0)	7,6 (6.2 to 8,5)	0.785	0.808	0.715
**Age 1^st^ exam**	8,5 (1,5 to 10,9)	8,5 (1,0 to 10,7)	8,5 (3,3 to 10,6)	9,0 (6.9 to 11,0)	0.196	0.819	0.187
**BA-CA (years)**	1,7 (−1,8 to 4,3)	1,4 (−,5 to 2,9)	0,8 (−1,4 to 6,1)	1,5 (−1,0 to 3,1)	0.861	0.347	0.933
**Height (SDS)**	1,0 (−3,2 to 4,8)	0,6(−1,7 to 2,9)	0,1 (−2,1 to 2,8)	1,1 (−1,7 to 2,2)	0.938	**0.047**	0.952
**BMI (SDS)**	1,0 (−2,7 to 4,9)	1.0 (−1,9 to 2,7)	1,1 (−0,4 to 3,5)	1,3 (0.1 to 2,8)	0.408	0.635	0.421
**E2 (pmol/L)**	42 (18 to 427)	37 (18 to 1052)	40 (18 to 401)	53 (18 to 611)	0.198	0.518	0.203
**Basal LH (IU/L)**	0,3 (0,05 to 9,4)	0,1 (0,05 to 15,3)	0,8 (0,05 to 6,7)	1,2 (0,06 to 5,5)	**0.023**	**0.031**	**0.025**
**Peak LH (IU/L)**	7,5 (0,2 to 77.9)	5,6 (1,7 to 21.1)	12,7 (1,0 to 50,1)	13.3 (1,6 to 63,3)	0.084	**0.043**	0.068
**LH/FSH ratio**	1,0 (0,1 to 9,8)	0,7 (0,2 to 2.1)	1,5 (0,1 to 6,8)	1,9 (0,3 to 3,72)	0.082	0.086	0.066

BA-CA: Bone age - Chronological age.

Basal LH was significantly higher in girls with newly diagnosed CNS pathology both comparing to normal scans only and to all non-pathological scans (p = 0.023 and p = 0.025, respectively). Furthermore, both basal LH and peak LH levels were significantly higher comparing all girls with pathological MRI scans(both newly diagnosed and known at time of referral) to all girls with non-pathological MRI scans (p = 0.031 and p = 0.043, respectively) (see Table 3). Height, expressed as SDS, was significantly lower in girls with pathological MRI compared to girls with non-pathological MRI (p = 0.047). No other parameters showed statistical differences. In terms of ethnicity 131 girls were of Danish origin, 37 were immigrants, 41 foreign-adopted and 20 had no information of ethnic origin in the patient file. In the group of girls with newly diagnosed CNS pathology two girls (ID 1 and 10, Table 2) were adopted and another two girls (ID 9 and 12, Table 2) were immigrants. Three girls who immigrated and one adopted girl had known CNS aetiology at time of referral.

A total of 104 girls had true precocious puberty according to international criteria (B2≤8 years and a pubertal GnRH response), out of whom 6 girls had OCPP based on newly diagnosed pathology and further 6 had OCPP based on known aetiology at time of referral. Median age at onset was 7.6 years (0.4 to 9.1) and 7.0 years (6.2 to 7.6) for girls with idiopathic CPP (ICPP) and newly diagnosed OCPP respectively, but this difference did not reach statistical significance (p = 0.058).

## Discussion

In this single-center cohort of 229 consecutive girls with early or precocious puberty, 34 had pathological CNS findings. Out of these, 21 were known CNS pathologies at time of referral. Importantly, 13 out of 208 girls with central precocious puberty and no other CNS symptoms presented with CNS pathologies upon MRI which were causally related to CPP. Another 20 girls presented with incidental findings.

Based on the MRI findings of pathological abnormalities associated to CPP from other studies, we know that some of the most common abnormalities include hypothalamic hamartomas, optic gliomas and arachnoid cysts, but the overview also shows that many other pathologies are associated to CPP (see overview in Table 4). We found 6 girls presenting with arachnoid cysts, one diagnosed prior to time of referral. The mechanism by which arachnoid cysts induce central activation of the hypothalamic-pituitary-gonadal axis remain unknown, but it has been suggested that the hypothalamic area is especially sensitive to compression, which also explains how hydrocephalus may cause CPP [11], [26], [27]. Four girls presented with astrocytomas, three being known at time of referral, which accounts for 12% of the CNS pathologies. Two girls presented with optic gliomas and NF1, one having hamartomas in capsula interna as well. Optic gliomas are known to cause CPP. Precocious puberty is frequently seen among patients with NF1 and optic pathway tumours [28]. Furthermore, NF1 is associated with hamartomas which were found in two girls with known pathology at time of referral. One girl presenting with precocious puberty without any other CNS symptom was diagnosed with NF1 after MRI findings of hamartomas and the finding of cafe au lait spots at clinical examination. Hamartomas are non-neoplastic heterotopias that initiates the rise in GnRH, and the malformation is a well known aetiology of precocious puberty, although the exact mechanism is still unidentified [10], [11]. Surprisingly, only one girl in our cohort was diagnosed with hypothalamic hamartomas, which contrasts a number of other studies [8], [16], [29].

**Table 4:** Summary of studies of precocious puberty.

Study	Year	Total	all patologies	Hamartomas	Astrocytomas/Gliomas	Arachnoid cysts	Other	Incidental findings	Organic precocpis puberty (%)
Bridges et al.	1994	91	6*	1		1	4		7
Chalumeau et al. (pilot)	2002	197	11	6	3	1	1		6
Chalumeau et al. (validation)	2002	42	3	3					7
Chalumeau et al.	2003	443	35	23	7	2	3	9	8
Chematily et al.	2001	230#	11	6	3	1	1		5
Choi et al.	2007	45	11	5	4		2		24
Cisternino et al.	2000	428	17	7	4	3	3	9	4
Kornreich et al.	1995	51	11	4	1		6		22
Ng et al.	2003	67	9	6	1	1	1		13
Soriano-Guilén et al.	2010	226	23	11			12		10
Taher et al.	2004	43	4	2		1	1	2	9
Mogensen et al.	2011	208#	13	2	1	5	5	20	6
Studies of girls with known pathologies									
Rivarola et al.	2001	12	12	4	3	4	1		
Trivin et al.	2006	15	15	10	3	1	1		
Trivin et al.	2006	52	52		20	5	27		
Mogensen et al.	2011	21	21	3	3	1	14		

*Previously diagnosed,

# only newly diagnosed.

We found 20 girls presenting with incidental findings on their brain MRI, which were not considered related to early pubertal development. Such findings, although incidental, usually cause considerable parental concern, but also in many cases repeated MRI scans. More than half the girls in our study had at least two control scans, which include general anaesthesia in some cases. Incidental findings are reported in other studies [5], [8], [30], but the psychological impact on the involved girls and their parents is, although well known to clinicians, not reported in publications to our knowledge. Unfortunately, there are currently no predictive clinical or biochemical parameters that could assist clinicians in avoiding this dilemma.

### Predictive factors in our study

Basal LH levels, but not the GnRH stimulated LH/FSH ratio, were significantly higher in girls with newly diagnosed CNS pathology compared to girls with a normal brain MRI. Previously we have found that basal LH levels is the superior biochemical parameter to predict the outcome of a GnRH test [4]. This could imply that the progression of puberty is faster in girls with organic cause, although this is speculative. Likewise, serum estradiol levels in girls were higher compared to girls with a normal MRI in agreement with previous studies [8] although the difference did not reach statistical significance.

True precocious puberty is traditionally defined by pubertal onset before 8 years of age in girls. Our recent data from Denmark showing a downwards secular trend in age at onset of breast development could theoretically question the appropriate cut off ages for performing a brain MRI [16]. Furthermore, the recent data from US illustrates that a high proportion of otherwise healthy 6–8 year old girls present with breast development [17]. However, in the Danish study we found that the early breast development was not accompanied by pituitary-gonadal activation, and therefore concluded that the early-pubertal girls were more likely to represent a thelarche-variant, which obviously do not need a brain MRI. As our data is based on a Danish population, it may not necessarily apply to other populations. On the other hand our present data clearly illustrates that brain MRI abnormalities occur in girls referred with premature sexual maturation in the 6–8 year age groups (and even in the 8–9 year age group), especially in girls with rapidly progressive puberty and biochemical signs of pituitary-gonadal activation. Thus, we believe it would be wrong to change current recommendations of brain MRI in girls with CPP before age 8, based on the finding of an increasing incidence of a pubertal thelarche-variant in population-based studies [16]. The typical clinical presentation encompass a continuum from an early-normal and slowly progressing pubertal variant to the rapidly progressing variant. Clinical follow up is there mandatory before a decision on which patients to perform GnRH testing and brain MRI can be taken.

Even though not statistically significant, age at pubertal onset was still lower in girls with newly diagnosed OCPP compared to girls with ICPP, which supports the hypothesis that age at pubertal onset may be a positive predictor of OCPP. Using the diagnostic tree presented by Chalumeau et al. [14], we would have missed three girls with CNS pathology aged 6–8 years; one requiring surgery (ID 3,4 and 6, Table 2). Interestingly, of the 68 girls who had MRI performed with pubertal onset after 8 years of age, six girls had newly diagnosed CNS pathology, none older than 8.5 years.

These data are unique as most other studies include girls only up to 8 years [5]–[10], [14], [16], [29]–[32]. Our results suggest caution when using medical history as criteria for performing MRI, as also girls older than 8 years at pubertal onset can present with pathological brain abnormality.

It has previously been reported that foreign-adopted children coming to Denmark has increased risk of precocious puberty [33]. In our study, even though finding 3 girls with CNS pathology, the percentage of pathology is lower in foreign-adopted girls (7.3%) than in girls with Danish origin and immigrants (15.3% and 13.5%, respectively). We believe that foreign-adoption could be a negative predictor for CNS pathology, but further studies must be made until making any final conclusions on this matter.

In conclusion, 13 out of 208 girls (6.3%) with early or precocious puberty and no other CNS symptom had pathological CNS findings upon MRI, which were causally related to CPP. Importantly, all 13 girls were above 6 years of age, and 6 girls were even 8–9 year old. A high frequency of our 6–8 year old girls with early or precocious had a pathological brain MRI, which could not be predicted from any clinical or biochemical parameters in our study. Thus, we believe that girls with precocious pubertal development of central origin before 8 years of age should continue to be screened by brain MRI.
